# A genetically unique Chinese cattle population shows evidence of common ancestry with wild species when analysed with a reduced ascertainment bias SNP panel

**DOI:** 10.1371/journal.pone.0231162

**Published:** 2020-04-09

**Authors:** Mario Barbato, Michael P. Reichel, Matilde Passamonti, Wai Yee Low, Licia Colli, Rick Tearle, John L. Williams, Paolo Ajmone Marsan

**Affiliations:** 1 Department of Animal Science, Food and Technology–DIANA, and Nutrigenomics and Proteomics Research Center–PRONUTRIGEN, Università Cattolica del Sacro Cuore, Piacenza, Italy; 2 Jockey Club College of Veterinary Medicine and Life Sciences, City University of Hong Kong, Kowloon, Hong Kong; 3 School of Animal and Veterinary Sciences, Davies Research Centre, University of Adelaide, Roseworthy, Australia; 4 Research Center on Biodiversity and Ancient DNA–BioDNA, Università Cattolica del Sacro Cuore, Piacenza, Italy; Universita degli Studi di Pavia, ITALY

## Abstract

In Hong Kong, there is a cattle population of ~1,200 individuals of uncertain origin and genetic diversity. This population shows heterogeneous morphology, both in body type and pigmentation. Once used as draught animals by the local farmers, they were abandoned around the 1970s due to changes in the economy, and since then have lived as feral populations. To explore the origins of these cattle, we analysed ~50k genotype data of 21 Hong Kong feral cattle, along with data from 703 individuals of 36 cattle populations of European, African taurine, and Asian origin, the wild *x* domestic hybrid gayal, plus two wild bovine species, gaur and banteng. To reduce the effect of ascertainment bias ~4k loci that are polymorphic in the two wild species were selected for further analysis. The stringent SNP selection we applied resulted in increased heterozygosity across all populations studies, compared with the full panel of SNP, thus reducing the impact of ascertainment bias and facilitating the comparison of divergent breeds of cattle. Our results showed that Hong Kong feral cattle have relatively high levels of genetic distinctiveness, possibly due to the low level of artificial selection, and a likely common ancestry with wild species. We found signs of a putative taurine introgression, probably dating to the import of north European breeds during the British colonialism of Hong Kong. We showed that Hong Kong feral cattle, are distinct from *Bos taurus* and *Bos indicus* breeds. Our results highlight the distinctiveness of Hong Kong feral cattle and stress the conservation value of this indigenous breed that is likely to harbour adaptive genetic variation, which is a fundamental livestock resource in the face of climate change and diversifying market demands.

## Introduction

Livestock domestication started ~12,000 years ago (YA) and marked the most significant transition in human history, providing nutrients, traction, fertilizer, leather, fuel and provisions. To date, more than 8,800 breeds of livestock have been reported. However, the actual number of extant breeds is likely to be larger as a significant proportion of indigenous livestock populations, which are present in the developing world, have yet to be described at genotypic and phenotypic level [1,2]. Indigenous domestic populations (often referred to as native breeds) are typically unmanaged genetically, or managed through traditional husbandry. These native breed generally show high levels of phenotypic variation [3–7] and are better adapted to local environments than specialised dairy and beef breeds, which are mostly of European taurine origin [8–10]. Indigenous populations have been reported for the major livestock species, including sheep, goat, pig, and cattle [7,11–13].

Cattle are among the most important livestock species worldwide, with almost ~1.5 billion individuals in 2012, of which almost a quarter were present in India and China [2]. Two main cattle domestication events have been described, the first ~10,000 YA in the Fertile Crescent, which gave rise to taurine cattle (*Bos taurus*), and the second ~2,000 years later in the Indus Valley, giving rise to indicine cattle (*Bos indicus*) [14,15]. After domestication, cattle were spread across the world following human migration and trade. In particular, taurine cattle spread into Europe, Africa, and Central and North-East Asia, following agriculture expansion [16]. Indicine cattle moved into South East Asia ~3,000 YA and Africa ~4,000 YA, where they were crossed with local taurine cattle to create Sanga populations [16–18]. Introgression between taurine and indicine genomes was also observed in Central Asia, along the fringe of colonization of *B*. *indicus* in the South and *B*. *taurus* in the North, and in Southern Europe where genomic analyses have identified components of both African taurine and indicine ancestry in several breeds [19,20].

**Fig 1:**
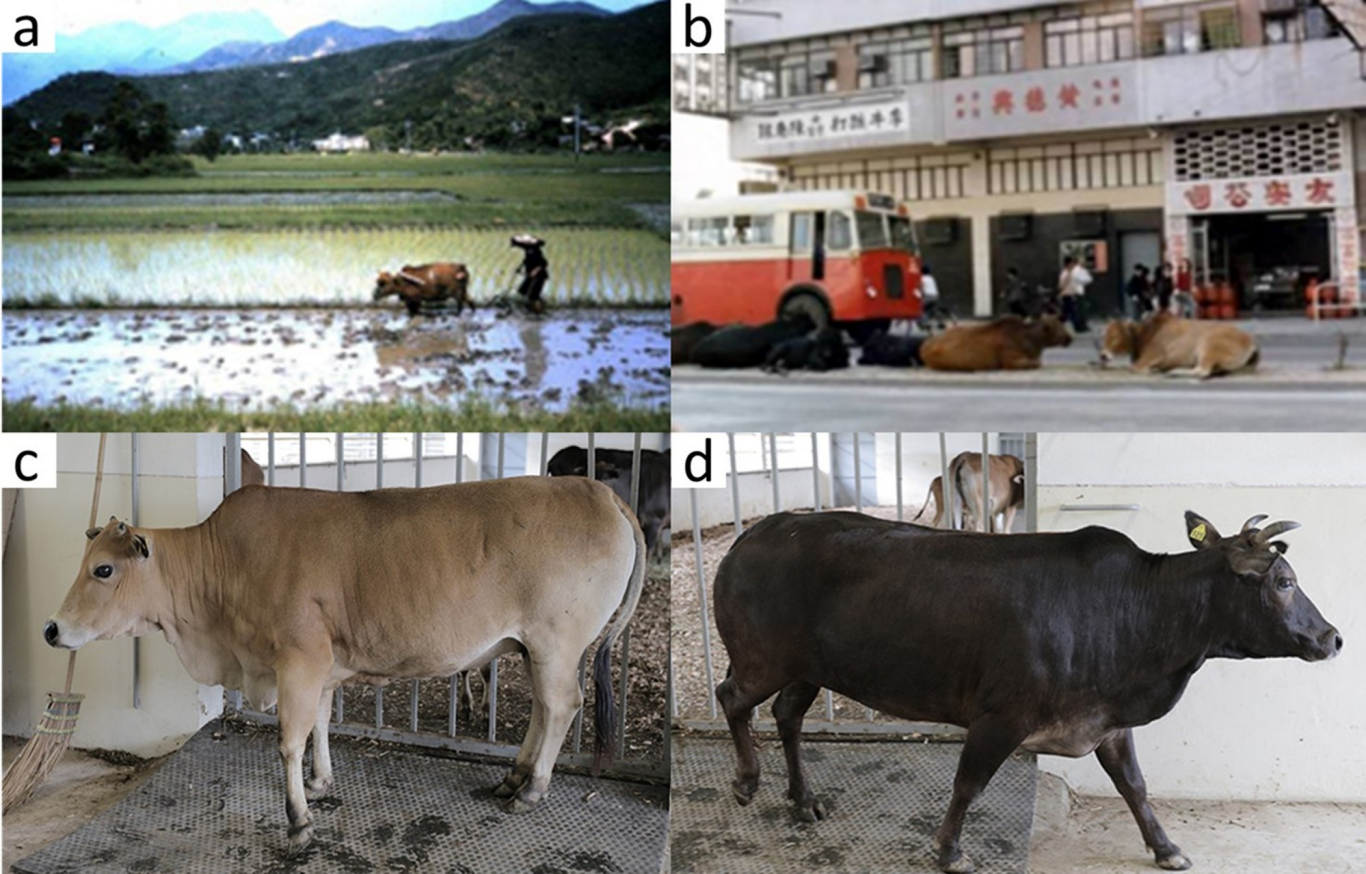
Hong Kong feral cattle. A) Local cattle used as draught power in Yuen Long field in Hong Kong, ca.1960, and B) wandering in Yuen Long Dam Road Museum, ca.1950. C-D) Hong Kong feral cattle animals from the herd of Twa Ku Ling government farm (Photographs: Thomson MUI).

A cattle population of over 1,200 head exists in the proximities of Hong-Kong and Southern China [21], but little is known of its ancestry or genetic diversity, with only anecdotal reports suggesting a relationship with Indonesian wild cattle (S1 Text). Prior to 1970s cattle were widely used by local farmers as draught animals, but as the economy shifted towards service industries the cattle were abandoned. Currently, the Hong Kong cattle survive as feral populations in the less urbanised areas of the city and are characterised by substantial phenotypic heterogeneity in terms of horn shape and orientation, coat colour, hump size and shape, tail length, and conformation (Fig 1).

To date no genetic assessment has been undertaken of Hong Kong feral cattle. Here we used genomic tools to investigate the genetic diversity of Hong Kong feral cattle (HKF) and compared them with taurine, indicine, and composite cattle breeds.

## Materials and methods

### Samples and genotyping

Hair samples of 21 HKF individuals of both sexes were collected in the Chong Hing in Sai Kung Country Park and the Ta Kwu Ling Government Farm. The procedure followed the specific conditions of a licence granted under the Animals (Control of Experiments) Ordinance Chapter 340 the Laws of Hong Kong (Licence Nos (16–47 to 50) in DH/HA&P/8/2/5 Pt 5) and with City University of Hong Kong Ethical Committee approval. Animals sampled are part of population of approximately 1,200 head of feral cattle located mainly on Lantau Island, Sai Kung and the East and North New Territories of Hong Kong [21]. DNA was extracted from hair follicles using a commercial kit (PureLink™ Genomic DNA Mini Kit from ThermoFisher) according to the manufacturer’s instructions and was genotyped with the Illumina BovineSNP50 v2 BeadChip array. For comparison, we collected genotypes of 703 individuals from 36 cattle breeds, including ten European taurine and two African taurine, two African Sanga, four Asian indicine breeds, 15 Asian local breeds including one Indonesian and 14 cattle breeds from Central and Southern China, two Asian wild *Bos* species: gaur (*Bos gaurus*) and banteng (*Bos javanicus*), and the semi-wild gayal (*Bos frontalis*), obtained from public repositories [19,22–25].

To ensure a high-quality genotype dataset, only loci with less than 10% missing data and minor allele frequency (MAF) higher than 1% were used. Loci with unknown map position or located on the sex chromosomes were also removed. Genotype data filtering was performed using Plink v1.9 [26]. As the Illumina 50k SNP panel was selected from genome sequences of taurine origin the SNP are predominantly those commonly found in taurine breeds [27,28]. Such a bias is proportional to the degree of divergence between the discovery and the study populations [29] and affects single locus statistics more than multilocus or haplotype-dependent analyses [29–31]. To reduce the impact of ascertainment bias in this study we used the following approach. First those SNPs which are polymorphic in banteng or gaur were selected. We did not use the hybrid gayal, which is a gaur *x* zebu hybrid [32]. As the divergence time between taurine cattle and banteng with gaur is 2.6 and 3 million years, respectively [33], polymorphisms shared between either of these two wild species and domestic cattle are likely to be ancestral rather than new variations that occurred after species divergence. Then, we pruned the SNPs with high values of linkage disequilibrium (LD) from the ancestral SNP set, which has been shown to reduce the impact of ascertainment bias [31]. LD pruning was performed using Plink (--indep-pairwise 2000kb 10 0.2).

### Genetic diversity and population structure

Heterozygosity (*H*_*o*_) and inbreeding (*F*) coefficients were computed using the R-Bioconductor package snpStats v1.30.0 [34]. To evaluate the ascertainment bias reduction, 1) the same genetic diversity analyses were performed using the whole dataset and the set of ancestral polymorphic loci identified in the wild cattle species, 2) the non-random effect of the ancestral SNPs on *H*_*o*_ was assessed through resampling by permutation. In brief, we sampled without replacement the same number of SNPs as our ancestral SNP set and computed the heterozygosity at the population level; this algorithm was run 1,000 times and the distribution of *H*_*o*_ obtained for each population was compared with the same index computed using the full and ancestral SNP set. A *p*-value was calculated for each *H*_*o*_ result obtained using the ancestral panel following Davison and Hinkley [35]: *pval = (1 + r)/(1 + n)*, where *r* is the number of permutations that produced an *H*_*o*_ value greater than or equal to that calculated for the ancestral panel and *n* is the total number of permutations.

Unsupervised hierarchical clustering analysis of population structure was performed using ADMIXTURE v1.23 [36]. The number of clusters computed ranged *K* values from 2 to 10. Supervised clustering analyses were carried out using ADMIXTURE with prior population information for European taurine (ANG, HOL, HFD, both as a meta-group and separating each breed), African taurine (MUT and non-admixed NDA individuals), indicine (GIR, THA, LOH), and banteng, gaur and gayal. Admixture plots were generated using AIDmixture v0.1 (https://github.com/barbatom/AIDmixture). To investigate the ordinal relationships between populations and individuals, model-free clustering was performed using principal component analysis (PCA) as implemented in Plink; a PCA on the HKF population alone was also performed. A Neighbour-net graph using Reynolds’ distances, calculated with a custom script, was generated using SplitsTree v4.13.1 [37].

The occurrence of gene flow was further investigated using Treemix v1.12 [38]. This software models the relationship among the sample populations and their ancestral population using genome-wide allele frequency data and a Gaussian approximation of genetic drift [38]. First, we generated maximum-likelihood-based phylogenetic tree of all cattle populations, and iteratively, we added one migration edge to the previously generated graph with “m” migration edge [20]. We rooted the graphs using banteng as outgroup. Up to 20 possible gene flow vertices were computed and the proportion of information added by each ‘m’ was assessed using the *f* statistics as implemented in Treemix. All graphs were generated using the statistical software R [39]. All data generated during this study are available as supplementary material (S1 Dataset).

## Results

After pruning for missing data, MAF and unmapped variants 31,482 autosomal SNPs were retained, of these 3,812 SNPs were polymorphic in either banteng or gaur, and were selected as the ancestral SNP dataset. Using this ancestral SNP dataset *H*_*o*_ in the HKF was 0.268. Among the European taurine breeds, *H*_*o*_ ranged from 0.305 to 0.345, with Hereford and Piedmontese having the lowest and highest values, respectively (Table 1). Heterozygosity for indicine breeds ranged from 0.227 in Tharparkar to 0.264 in Nellore (Table 1). African taurine had *H*_*o*_ values from 0.227 to 0.283, and Sanga breeds from 0.322 to 0.342. Asian local breeds from Central/Northern China had heterozygosity values ranging from 0.340 (Enshi) to 0.370 (Lhasa), whereas populations sampled from southern China and Indonesia had lower values ranging from 0.263 (Aceh) to 0.355 (Honghe). The wild and wild-hybrid species had the lowest *H*_*o*_ values: 0.183 (Gayal), 0.132 (Banteng) and 0.112 (Gaur).

**Table 1:** Sample information and diversity indexes. Breed/population name, acronym, cattle type, and number of individuals analysed in this work are shown in the first four columns. Observed heterozygosity (*H*_*o*_), inbreeding coefficient (*F*) and the corresponding standard deviations (*SD*) were calculated using the 3,812 SNP ancestral data set.

Breed	Acronym	Type	Number	*H*_*o*_ *(SD)*	*F (SD)*
Angus	ANG	European taurus	24	0.314 (0.02)	0.21 (0.06)
Hereford	HFD	European taurus	24	0.305 (0.04)	0.24 (0.11)
Brown Swiss	BSW	European taurus	24	0.309 (0.01)	0.23 (0.03)
Fleckvieh	FLV	European taurus	24	0.328 (0.01)	0.18 (0.02)
Holstein	HOL	European taurus	24	0.332 (0.01)	0.17 (0.03)
Limousine	LMS	European taurus	24	0.330 (0.01)	0.17 (0.04)
Piedmontese	PIE	European taurus	24	0.345 (0.01)	0.14 (0.02)
Romagnola	ROM	European taurus	24	0.321 (0.01)	0.20 (0.03)
Marchigiana	MCG	European taurus	13	0.329 (0.02)	0.18 (0.04)
Chianina	CHI	European taurus	16	0.316 (0.01)	0.21 (0.02)
Muturu	MUT	African taurus	13	0.227 (0.01)	0.43 (0.03)
N'Dama	NDA	African taurus	23	0.283 (0.05)	0.29 (0.12)
Nganda	NGA	Sanga	24	0.342 (0.02)	0.14 (0.04)
Ankole	ANW	Sanga	24	0.322 (0.01)	0.19 (0.02)
Gir	GIR	Indicus	24	0.260 (0.01)	0.35 (0.03)
Tharparkar	THA	Indicus	13	0.227 (0.02)	0.43 (0.06)
Lohani	LOH	Indicus	13	0.252 (0.03)	0.37 (0.07)
Nellore	NYP	Indicus	24	0.264 (0.01)	0.34 (0.02)
Yanbian	YAB	Asian local N	24	0.323 (0.02)	0.19 (0.04)
Lhasa	LHS	Asian local N	14	0.370 (0.02)	0.07 (0.05)
Linzhi	LIZ	Asian local N	19	0.358 (0.02)	0.10 (0.05)
Qinchuan	QIC	Asian local N	24	0.365 (0.01)	0.08 (0.04)
Jinnan	JIN	Asian local N	14	0.363 (0.02)	0.09 (0.05)
Aceh	ACE	Asian local S	12	0.263 (0.01)	0.34 (0.02)
Banna	BNA	Asian local S	14	0.301 (0.03)	0.25 (0.07)
Dehong	DEH	Asian local S	16	0.273 (0.03)	0.32 (0.08)
Honghe	HOH	Asian local S	12	0.355 (0.02)	0.11 (0.04)
Dengchuan	DEC	Asian local S	24	0.338 (0.02)	0.15 (0.05)
Luxi	LUX	Asian local S	11	0.363 (0.01)	0.09 (0.02)
Nanyang	NAY	Asian local S	23	0.357 (0.01)	0.11 (0.02)
Enshi	ENS	Asian local S	24	0.340 (0.02)	0.15 (0.06)
Wannan	WAN	Asian local S	24	0.319 (0.02)	0.20 (0.05)
Wenling	WEL	Asian local S	24	0.295 (0.03)	0.26 (0.07)
Hong Kong feral	HKF	Feral	21	0.268 (0.02)	0.35 (0.05)
Gayal	OGA	Semi-Wild	21	0.183 (0.05)	0.54 (0.13)
Banteng	BAN	Wild	14	0.132 (0.01)	0.67 (0.04)
Gaur	GAU	Wild	10	0.112 (0.01)	0.72 (0.02)

HKF had an inbreeding value *F* = 0.350, which was comparable with indicine breeds *F* values ranging from 0.340 (Nellore) to 0.430 (Tharparkar). Most of the European taurine had lower *F* values, with Hereford having the highest and Piedmontese the lowest (0.240 and 0.140 respectively; Table 1). Asian local populations showed great variation in inbreeding values, which were lower among the Central/Northern China populations (*F* = 0.07–0.11), and higher among the southern China and Indonesian samples (*F* = 0.19–0.35).

We compared the genetic diversity results obtained in the ancestral dataset (~4k SNPs) with those obtained using the whole dataset (~32k SNPs) to evaluate the reduction in ascertainment bias. When the ancestral dataset was used, *H*_*o*_ values for each cattle type were higher than those obtained with the full dataset (see S1 Table). The HKF, indicine, local Chinese and wild populations showed the greatest increase in heterozygosity with respect to the full dataset, with an increase in median *H*_*o*_ values three-fold higher than that obtained for taurine-breeds, whereas median *H*_*o*_ for Sanga and Asian local breeds increased in two-fold (S1 Table and S2 Fig). We compared the *H*_*o*_ values obtained using the full and ancestral SNP panels with the distribution of *H*_*o*_ values obtained through 1,000 permutations of the same number of SNPs as in the ancestral set (S3 Fig). The *H*_*o*_ values obtained using the ancestral panel were consistently higher than those obtained through permutation (*p*-value <0.001) except in Holstein, Angus and Hereford (*p*-value = 0.024, 0.007 and 0.005, respectively; S2 Table and S3 Fig). Conversely, the *H*_*o*_ computed using the full SNP panel had values close to the mean of the *H*_*o*_ distributions obtained from permutations (*p*-value >0.4; S2 Table).

**Fig 2:**
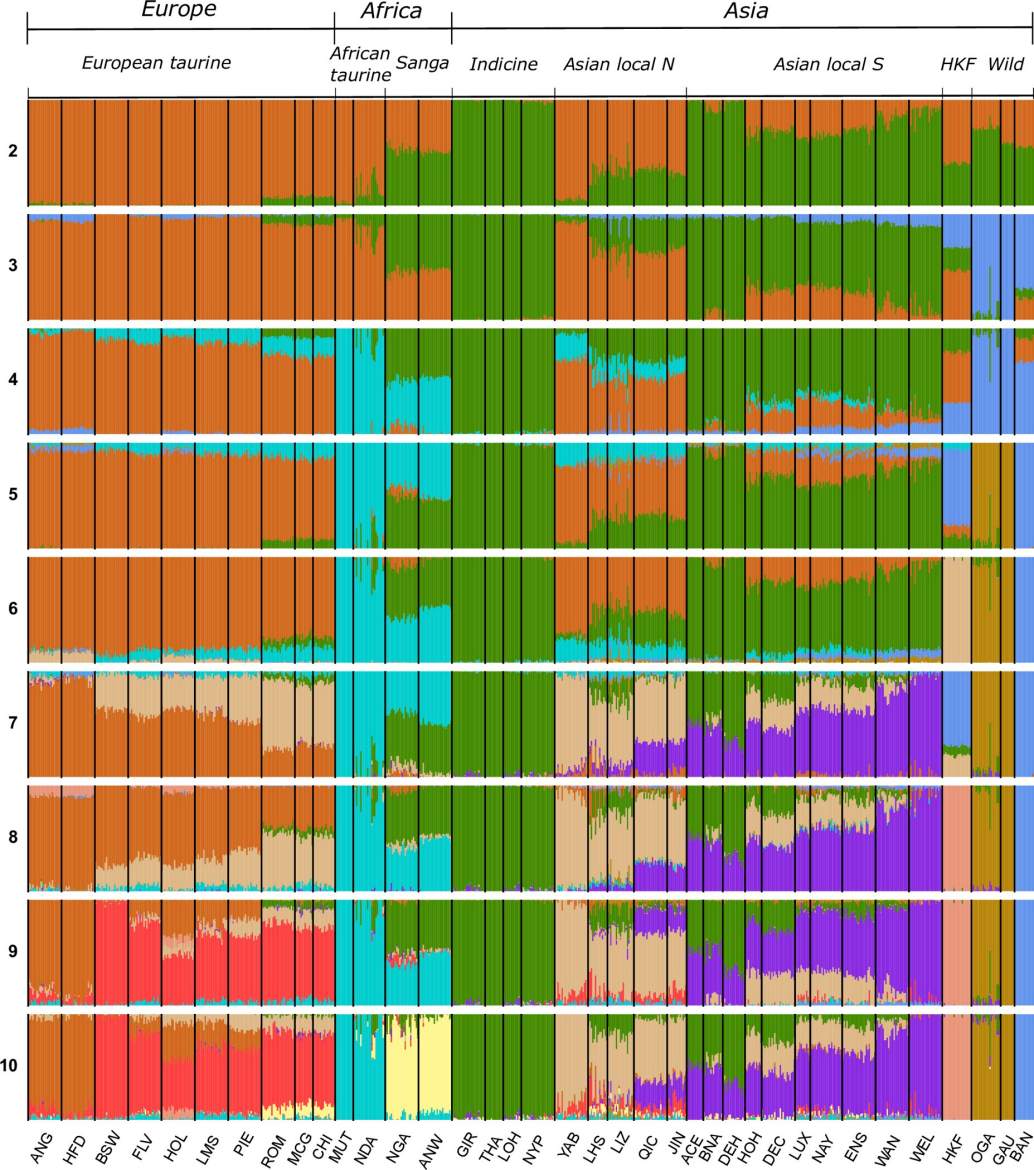
Admixture analysis of the first 10 *K* solutions for 37 cattle populations. For population abbreviations see Table 1.

Admixture analysis at *K* = 2 discriminated Asian ancestry (green in Fig 2), which was shared by indicine populations, Asian local cattle, the two Asian wild species, and European-African populations with taurine ancestry (brown in Fig 2).

At *K* = 2 Sanga, Asian local breeds, and HKF showed mixed proportions of Asian and taurine ancestral components. Among the Asian local breeds, those from north China had <30% Asian ancestry and the other Asian breeds showed >60%. Southern European breeds such as Chianina, Romagnola and Marchigiana, and the Yanbian from north-west China had around 10% Asian ancestry, whereas the two African taurine breeds (Muturu and N’Dama) were predominantly European-African, but showed some of the Asian component (Fig 2). At *K* = 3 the wild and the indicine populations were discriminated, whereas *K* = 4 separated the European and African taurine ancestry. At *K* = 4 HKF had ~30% of wild Asian ancestry, whereas the remaining ~70% was assigned to a combination of European taurine and Asian indicine ancestry (~60 and ~30%, respectively; Fig 2). A small amount of the cluster component characterising the wild Asian populations were present in the north European taurine breeds, specifically Hereford, Angus, and Holstein, and <10% of those characterising indicine and taurine breeds were found in banteng. Both Sanga breeds showed comparable proportions of African taurine and indicine ancestry; however, some European taurine ancestry was observed, particularly in Nganda. The African taurine N’Dama was predominantly African taurine but the 11 animals showed differing levels of indicine ancestry. African taurine ancestry was also seen in the majority of European breeds, particularly those from Southern Europe. At *K* = 5 gayal and gaur separated as a distinct group, with only a small amount of this ancestry present in Hereford, and to a lesser extent in Angus and Holstein. At *K* = 7 Asian local breeds (excluding Yabian) shared a genetic component (purple in Fig 2) which mostly characterised the south-east China population Wannan and Wenling (>90%; Fig 2). At higher *K* values HKF remained as a unique cluster (Fig 2), although a small proportion of the cluster component (<10%) is present at *K* = 8 in the north European breeds Holstein, Angus and Hereford, and at *K* = 10 this component is found exclusively in Holstein. Supervised admixture analysis assigned ~60% European taurine, ~20% indicine, ~10% gaur and ~10% banteng ancestry to HKF. The same analysis performed with each European taurine population as a separate prior population assigned ~100% Holstein ancestry to HKF (S4 Fig).

**Fig 3:**
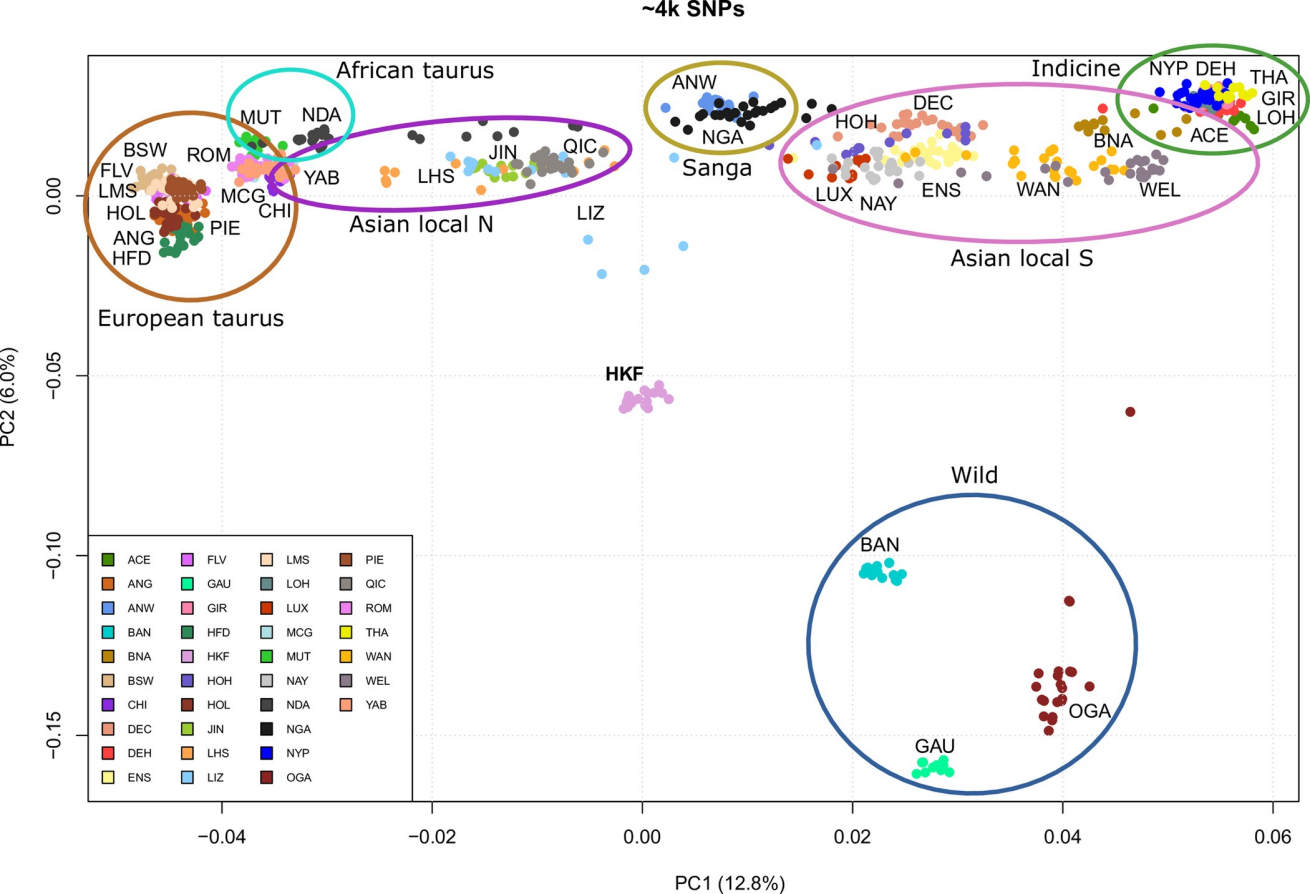
Principal component analysis. The percentage values within brackets refer to the proportion of variance explained by each of the displayed principal components. For population abbreviations see Table 1.

The first principal component (PC) of the PCA accounted for 12% of the variance and discriminated taurine and indicine origins (Fig 3, x-axis), mirroring Admixture results from *K* = 2. The second PC accounted for 6.8% of the variance and reflected Admixture results for *K* = 3, discriminating wild and HKF from other populations (Fig 3, y-axis). The first two PC combined identified six distinct clusters corresponding to the main cattle types analysed in this work (Fig 3, Table 1). HKF formed a distinct tight cluster in an intermediate position between indicine and taurine populations for the first PC and between the wild animals and the domestic cattle for the second PC.

**Fig 4:**
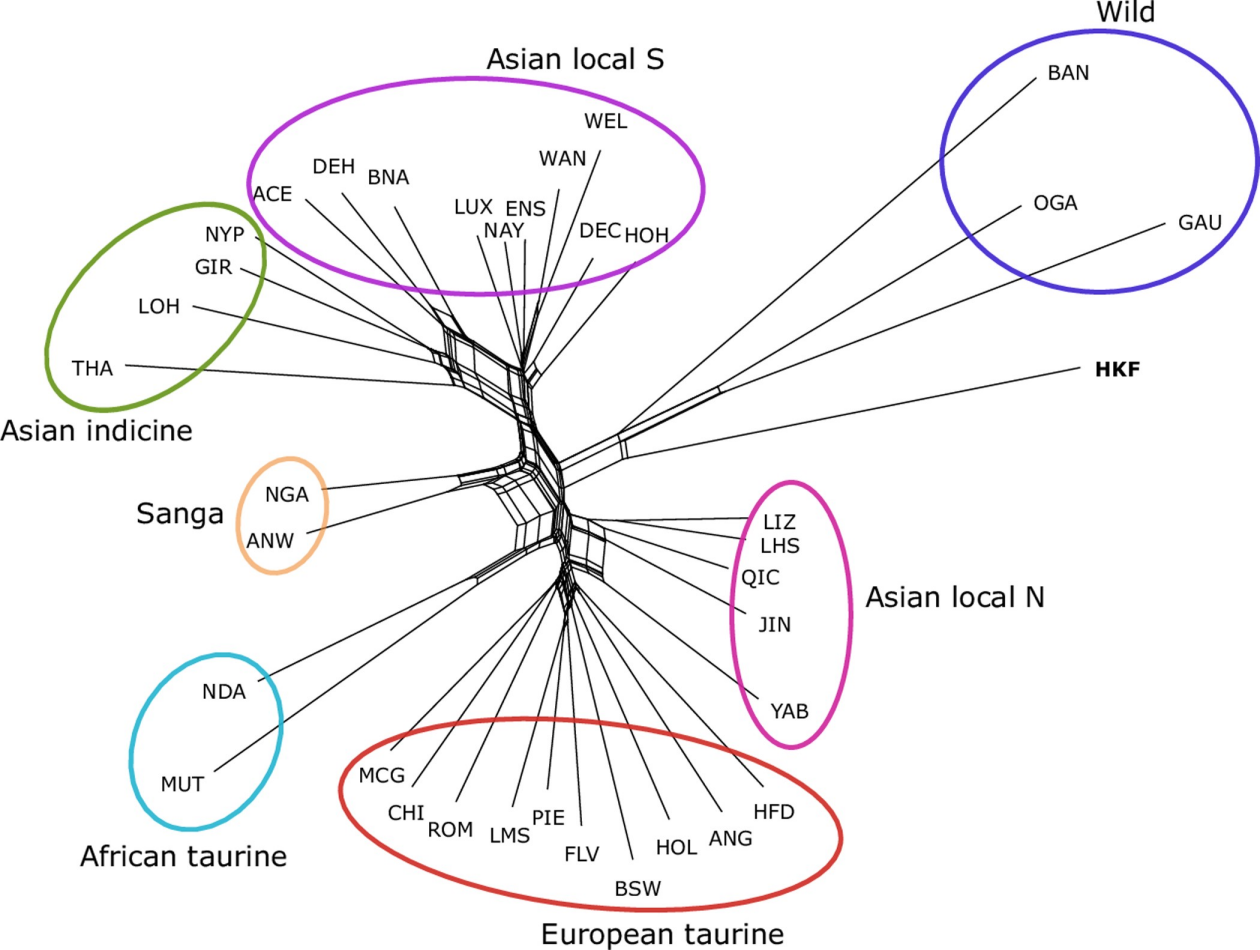
Neighbour-net of Reynold’s distances. For population abbreviations see Table 1.

The Neighbour-net analysis of pairwise Reynolds’ distances clearly discriminated among cattle types and geographic origin, with Asian indicine and European taurine breeds at opposite sides of the network (Fig 4), with the African breeds positioned in between. The pure African taurine breeds were closer to the European taurine branch and the crossbred Sanga were closer to the Asian indicine branch. The Asian local populations were split, such that the branch with the Northern breeds was closer to the European taurine group, and the remaining populations were located closer to the Asian indicine cluster. In particular, the Aceh, Dehong and Banna breeds showed more connections with the Asian indicine than the taurine group. The wild species and HKF clustered in a distinct branch, confirming the distinctiveness of HKF compared with other cattle, and closer genetic relationship of this population with the wild species.

**Fig 5:**
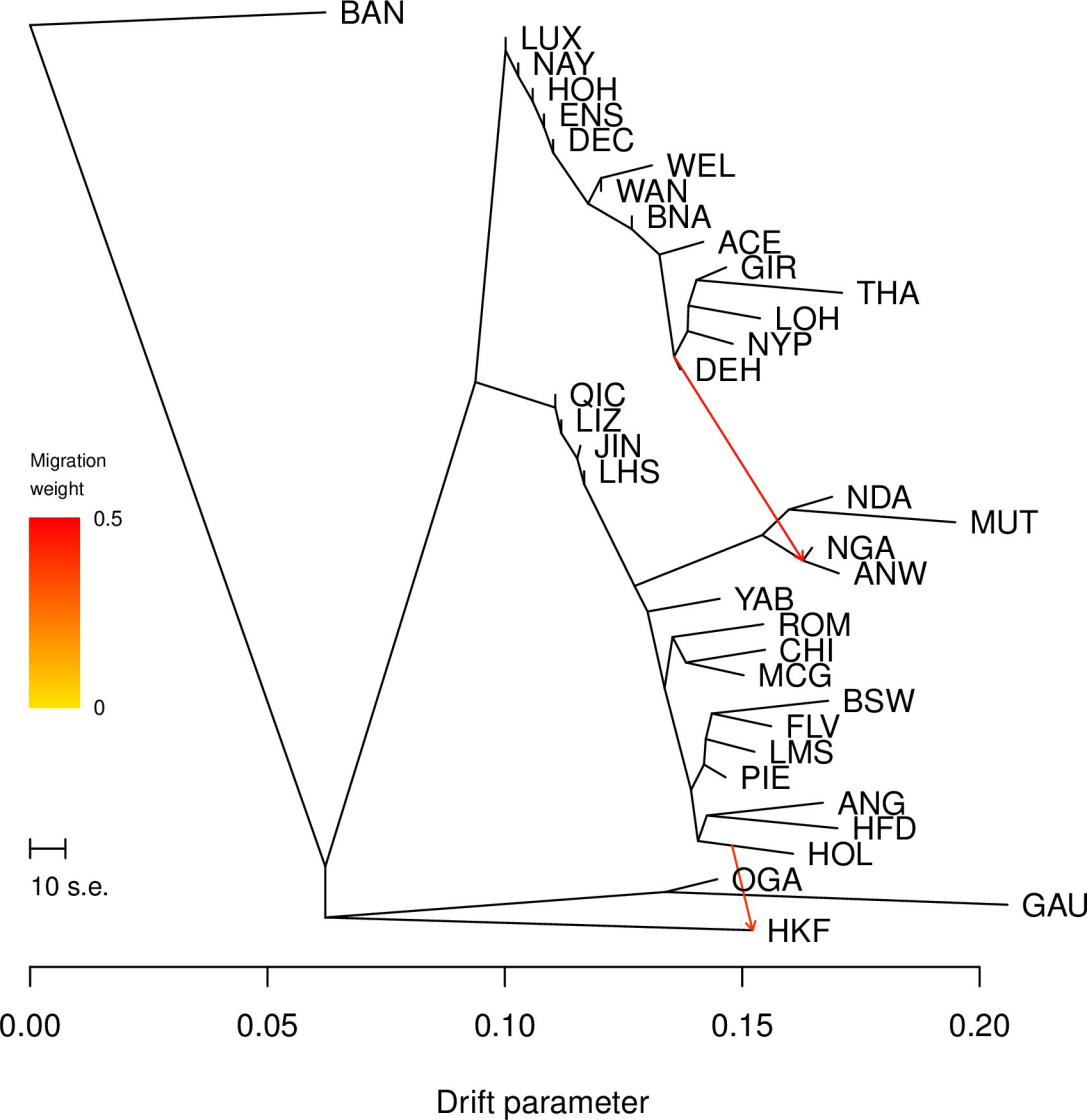
Treemix plot. Phylogenetic network inferred by Treemix of the relationships among the cattle populations in our dataset. Putative gene flow is identified by the arrows, pointing in the direction of the recipient population and coloured in red proportionally to the gene flow intensity.

A maximum likelihood assessment of population history with overlaid gene flow was performed using Treemix (Fig 5). From the *f* statistics the first migration edge was found to be the most informative, and suggested gene flow from the node representing the Asian indicine breeds to the node representing the Sanga breeds. The second—less informative—migration edge suggested gene flow from the Holstein to HKF (Fig 5).

## Discussion

We used genome-wide genotypic data to assess the genetic diversity and structure of a feral cattle population from the Hong Kong area, and compared it with breeds representative of worldwide cattle diversity, along with 15 local breeds and two wild *Bos* species from southern Asia. Our results showed that HKF had higher levels of genetic diversity compared with local cattle breeds from south Asia and most likely common ancestry or introgression from wild cattle.

### Reduction of ascertainment bias

The SNPs selected to develop the BovineSNP50 BeadChip were identified in taurine cattle breeds and it is likely that they underestimate diversity in non-taurine populations [27,40]. To reduce this ascertainment bias we selected ancestral polymorphisms that are present in banteng and gaur as well as cattle, and pruned the SNPs in high LD. Of the initial 48k SNP about 12% passed the initial quality filters leaving 32k high quality cattle SNP; of these ~4k SNP were polymorphic in the wild species and were not in LD (S5 Fig). A similar proportion (~10%) 50k SNP on the Illumina array was found to be polymorphic in bison, yak, or banteng [33]. The ancestral loci are less likely to show population bias, which is reflected in the higher heterozygosity of the reduced set of SNP in the non-taurine populations compared with that observed using the full dataset (S1 Table and S2 Fig) [31,40]. Using ancestral SNPs and pruning for LD has been shown to significantly reduce ascertainment bias in other studies [31], although fine-scale patterns of diversity among closely related breeds may be missed [31,41]. The data presented here, for the full SNP set, showed a decrease in heterozygosity proportional to the phylogenetic distance between the cattle breeds used for SNP discovery and the target population, whereas the reduced set significantly reduced this bias across all the populations studied (S1 Table and S2 and S6 Figs). As expected, when we tested the ability of the ancestral SNP panel to reduce ascertainment bias via resampling by permutation, the distance from the *H*_*o*_ computed using the ancestral set and the mean the *H*_*o*_ distribution for each bread was proportional to the phylogenetic distance between that breed and the taurine breeds used for SNP discovery (S3 Fig). Noticeably, some of the taurine breeds in our dataset, which were among those used in the 50k SNP chip discovery panel, recorded the highest *p*-values (S2 Table), confirming that the strategy implemented here removed those SNPs having the largest ascertainment bias impact. Importantly, the *H*_*o*_ values of the reference breeds obtained using our SNP selection approach were comparable with those of the Illumina BovineHD array, which is still today considered the most robust SNP array for cattle [24,42]. Further, the population structure analyses performed using the reduced dataset provided results in line with the know relationship among the reference populations in our dataset, whereas the same analysis performed using the full SNP was distorted by the percentage of taurine ancestry in a given breed (S6 Fig).

### Diversity and structure

We observed high levels of genetic variation in HKF, with values of heterozygosity comparable or higher than those seen for indicine cattle and the local breed from southern Asia, and only slightly lower than those of African and European taurine (Table 1).

Hybridisation between taurine and indicine breeds could have contributed to HKF diversity. Both taurine and indicine ancestry have contributed to Chinese cattle genetics, with Northern Chinese breeds having a higher taurine component than Southern breeds which have a greater indicine component (Figs 2–4) [22,43,44]. This is also reflected in mitochondrial data, [22,45–47]. The remarkable observation in the present study is that HKF are genetically distinct from pure taurine and indicine breeds and from indicus *x* taurine crossbred populations. We found no evidence of gene flow from any of the Asian local breeds to the HKF, despite the geographical proximity. The genetic divergence of the HKF from other cattle may be due to drift, resulting from a severe bottleneck or prolonged isolation, which would increase genetic distance from the original population. This type of founder effect has been identified in Chinese taurine cattle and a bottleneck in the indicine populations in the south-eastern part of China [22]. Another contributing factor could be geographical isolation. The Qinling Mountains traverse the Shaanxi province from east to west, creating a natural barrier between North and South-China which have restricted the expansion of taurine cattle southward and the spread of indicine northwards [46], and potentially isolated the Hong Kong cattle. However, we did not find any shared genomic ancestry between HKF and any of the Asian local populations.

Admixture analysis at *K* = 2–4, PCA, and Neighbour-net analyses identified the presence of wild, in addition to indicine and European taurine ancestry in the HKF (Figs 2–4). The second principal component clustered HKF, banteng, gayal and gaur separately from the other cattle populations (Fig 3), and the Neighbour-net analysis suggested that the HFK and the wild populations stemmed from the same independent branch (Fig 4). This supports the anecdotal records which suggest the contribution of wild bovine species to HKF. *B*. *javanicus* and *B*. *t*. *indicus* ancestry has been reported in several Asian cattle populations from genome-wide SNP and microsatellite analyses [19,48]. Introgression from banteng and gayal into domestic cattle breeds of Southern China has been identified from analysis of SNP and whole genome sequence [22,44]. Our analysis of admixture performed with Treemix positioned the HKF branch near the branches of the wild cattle and separated from the domestic cattle, but did not identify any evidence of gene flow from the wild species (Fig 5). Unsupervised admixture results at *K* = 5 and 7 suggest banteng as putative source of wild ancestry (Fig 2), whereas supervised admixture assigned equal proportions of banteng and gaur (S4 Fig). Hence, it is possible that the wild ancestry component to HKF sources from a different un-sampled wild population or it may carry admixture of more than one wild species [22,49].

Treemix and admixture analyses suggested a north European cattle contribution to HKF (Figs 2 and 5). This finding aligns with historic records mentioning that milk producing cattle from Holland where brought to Hong Kong around the time of the first British colonisation [50]. Supervised admixture analysis performed with the distinct north-European taurine breeds as prior ancestry clustered HKF exclusively with Holstein (S4 Fig). Although the association of HKF with Holstein aligns with Treemix and admixture results, the assigned ancestry proportion is clearly overestimated and possibly due to the reduced set of markers coupled with the restriction of the supervised model based assignment.

The diverse genetic origins of the HKF population would, at least partially, explain the phenotypic diversity among HKF population in which morphological traits span from zebu to taurine type (Fig 1).

### Population homogeneity

The HKF cattle are characterised by a striking phenotypic diversity, especially considering the genetic homogeneity (see Fig 1) seen by the tight PCA clustering and unique genetic profile from admixture analyses (Figs 2 and 3). The HKF PCA cluster showed no greater dispersion than highly selected cattle breeds. Low levels of breeding management have been associated with a large phenotypic variability in other indigenous cattle populations [3]. There was little phenotypic selection practiced for the HKF before being released to feral life which may explain the large phenotypic diversity seen [21]. However, the distinct genetics of this population is intriguing. It would be interesting to hypothesise that HKF are a remnant of a distinct cattle domestication. Indeed, recent results from whole genome sequence analyses of Asian cattle suggested the putative domestication of a genetically differentiated wild *B*. *t*. *indicus* population to explain the highly divergent ancestry of Southern Asian zebu breeds compared with other cattle populations [44].

## Conclusions

We applied stringent SNP selection to identify a panel of ~4k ancestral polymorphic loci to reduce the effect of ascertainment bias and facilitate the comparison of divergent breeds of cattle. Using this ancestral SNP set we identified the HKF population as genetically distinct from other taurine, indicine and crossbred cattle populations, and showed evidence of a significant contribution of wild bovine species to the genetics of the HKF. Further, we identified signals of putative introgression from north European cattle into HKF, possibly due to the import of high productive cattle during the British colonisation. Domesticated local breeds, such as HKF are likely to have adapted genetic variation to match the local environments as they have had low selection pressure for production traits. This is particularly important as globalization and productivity oriented breeding programs are homogenising the genetics and reducing variability among cattle populations. Preserving local genetic resources is therefore required to maintain a pool of variants which developed as a response to environmental pressures such as disease and parasite tolerance, heat tolerance, and adaptation to local feed resources. The loss of local breeds such as HKF will significantly reduce our ability to face and rapidly adapt to a changing environment. While showing that the HKF are genetically different from other cattle populations, additional unbiased data, including mtDNA, Y chromosome and whole genome sequences are necessary to better define the origins of the HKF cattle and explore whether they may be traced to an independent domestication event.

## Supporting information

S1 Text Additional historical information on HKF. (DOC)

S1 Dataset Genotype data of the HKF population. (ZIP)

S1 Table Diversity statistics computed on 37 cattle populations using ~32k and ~4k SNPs. (XLSX)

S2 Table Empirical *p*-values of population heterozygosity computed using the ancestral and full SNP panels (~4k and ~32k SNPS, respectively). (XLSX)

S1 Fig Geographical distribution of the cattle breeds included in the dataset. (PDF)

S2 Fig Comparison of heterozygosity distribution between the full (~32k SNPs) and ancestral (~4k SNPs) datasets stratified for cattle type. (PDF)

S3 Fig Comparison of heterozygosity values obtained using the full (~32k SNPs) and ancestral (~4k SNPs) datasets and the distribution of heterozygosity (coloured boxplots) computed with 1,000 permutations of the same number of SNPs as in the ancestral panel. (PDF)

S4 Fig Supervised Admixture analysis of HKF performed with ~4k SNPs using A) a meta-group of taurine references and B) separating the three European taurine breeds as prior populations. The European taurine (TaE) meta-group includes ANG, HFD and HOL, the African Taurine (TaA) includes MUT and the non-admixed individuals of NDA, and the Indicine (Ind) group includes GIR, THA and LOH.
(PDF)

S5 Fig Comparison of the distribution of the 3,812 and 31,482 SNPs selected for analysis in this work on the 29 bovine autosomes (chromosome number is on the left). The vertical lines in the upper and lower part of each chromosome schematic represent the SNPs in the ~4k and ~32k dataset, respectively.
(PDF)

S6 Fig A) Admixture analysis of the first 10 *K* solutions for 37 cattle populations using ~32k SNPs. B) Principal component analysis using ~32k SNPs. The percentage values within brackets refer to the proportion of variance explained by each of the displayed principal components. For population abbreviations see Table 1. C) Neighbour-Net of Reynold’s distances using ~32k SNPs.
(PDF)

## References

[1] FAO. COMMISSION ON GENETIC RESOURCES FOR FOOD AND AGRICULTURE: STATUS AND TRENDS OF ANIMAL GENETIC RESOURCES– 2018 [Internet]. 2019 Feb. Available: http://www.fao.org/3/my867en/my867en.pdf

[2] FAO. THE SECOND REPORT ON THE STATE OF THE WORLD’s FAO COMMISSION ON GENETIC RESOURCES FOR FOOD AND AGRICULTURE ASSESSMENTS • 2015 [Internet]. 2015. Available: www.fao.org/publications

[3] Giovambattista G, Ripoli M V., Peral-Garcia P, Bouzat JL. Indigenous domestic breeds as reservoirs of genetic diversity: the Argentinean Creole cattle. Anim Genet. John Wiley & Sons, Ltd (10.1111); 2001;32: 240–247. 10.1046/j.1365-2052.2001.00774.x 11683709

[4] Van Vuren D, Hedrick PW. Genetic Conservation in Feral Populations of Livestock. Conserv Biol. 1989;3: 312–317. 10.1111/j.1523-1739.1989.tb00091.x

[5] Bruford MW, Ginja C, Hoffmann I, Joost S, Orozco-terWengel P, Alberto FJ, et al Prospects and challenges for the conservation of farm animal genomic resources, 2015–2025. Front Genet. 2015;6: 314 10.3389/fgene.2015.00314 26539210 PMC4612686

[6] Oget C, Servin B, Palhière I. Genetic diversity analysis of French goat populations reveals selective sweeps involved in their differentiation. Anim Genet. 2019;50: 54–63. 10.1111/age.12752 30549070 PMC6590323

[7] Sermyagin AA, Dotsev A V., Gladyr EA, Traspov AA, Deniskova TE, Kostyunina O V., et al Whole-genome SNP analysis elucidates the genetic structure of Russian cattle and its relationship with Eurasian taurine breeds. Genet Sel Evol. BioMed Central; 2018;50: 37 10.1186/s12711-018-0408-8 PMC6042431 29996786

[8] Henriksen R, Gering E, Wright D. Feralisation—The Understudied Counterpoint to Domestication Origin and Evolution of Biodiversity. Cham: Springer International Publishing; 2018 pp. 183–195. 10.1007/978-3-319-95954-2_11

[9] Johnsson M, Gering E, Willis P, Lopez S, Van Dorp L, Hellenthal G, et al Feralisation targets different genomic loci to domestication in the chicken. Nat Commun. Nature Publishing Group; 2016;7: 12950 10.1038/ncomms12950 27686863 PMC5056458

[10] Pitt D, Bruford MW, Barbato M, Orozco-terWengel P, Martínez R, Sevane N. Demography and rapid local adaptation shape Creole cattle genome diversity in the tropics. Evol Appl. Wiley/Blackwell (10.1111); 2018; 10.1111/eva.12641 PMC6304683 30622639

[11] Deniskova TE, Dotsev AV, Selionova MI, Kunz E, Medugorac I, Reyer H, et al Population structure and genetic diversity of 25 Russian sheep breeds based on whole-genome genotyping. Genet Sel Evol. BioMed Central; 2018;50: 29 10.1186/s12711-018-0399-5 PMC5968526 29793424

[12] Iacolina L, Corlatti L, Buzan E, Safner T, Šprem N. Hybridization in European ungulates: an overview of the current status, causes and consequences. Mamm Rev. 2018; 10.1111/mam.12140

[13] Vajana E, Barbato M, Colli L, Milanesi M, Rochat E, Fabrizi E, et al Combining Landscape Genomics and Ecological Modelling to Investigate Local Adaptation of Indigenous Ugandan Cattle to East Coast Fever. Front Genet. 2018;9 10.3389/fgene.2018.00009 30333851 PMC6177531

[14] Loftus RT, MacHugh DE, Bradley DG, Sharp PM, Cunningham P. Evidence for two independent domestications of cattle. Proc Natl Acad Sci U S A. National Academy of Sciences; 1994;91: 2757–61. 10.1073/pnas.91.7.2757 8146187 PMC43449

[15] Pérez-Pardal L, Sánchez-Gracia A, Álvarez I, Traoré A, Ferraz JBS, Fernández I, et al Legacies of domestication, trade and herder mobility shape extant male zebu cattle diversity in South Asia and Africa. Sci Rep. 2018;8: 18027 10.1038/s41598-018-36444-7 30575786 PMC6303292

[16] Ajmone-Marsan P, Fernando Garcia J, Lenstra JA, The Globaldiv Consortium. On the Origin of Cattle: How Aurochs Became Cattle and Colonized the World. Evol Anthropol. 2010;19: 148–157. 10.1002/evan20267

[17] MacHugh DE, Shriver MD, Loftus RT, Cunningham P, Bradley DG. Microsatellite DNA variation and the evolution, domestication and phylogeography of taurine and zebu cattle (Bos taurus and Bos indicus). Genetics. 1997; 10.1093/genetics/146.3.1071 PMC1208036 9215909

[18] Hanotte O, Bradley DG, Ochieng JW, Verjee Y, Hill EW, Rege EO. African pastorialism: genetic imprintings of origins and migrations. Science (80-). 2002;296: 336–339. 10.1126/science.1069878 11951043

[19] Decker JE, McKay SD, Rolf MM, Kim J, Molina Alcalá A, Sonstegard TS, et al Worldwide Patterns of Ancestry, Divergence, and Admixture in Domesticated Cattle. McVean G, editor. PLoS Genet. Public Library of Science; 2014;10: e1004254 10.1371/journal.pgen.1004254 PMC3967955 24675901

[20] Upadhyay M, Bortoluzzi C, Barbato M, Marsan PA, Colli L, Ginja C, et al Deciphering the patterns of genetic admixture and diversity in southern European cattle using Genome-wide SNPs. Evol Appl. John Wiley & Sons, Ltd (10.1111); 2019; 10.1111/eva.12770 PMC6503822 31080507

[21] Massei G, Koon KK, Benton S, Brown R, Gomm M, Orahood DS, et al Immunocontraception for managing feral cattle in Hong Kong. PLoS One. 2015;10: 1–14. 10.1371/journal.pone.0121598 PMC4391848 25856283

[22] Gao Y, Gautier M, Ding X, Zhang H, Wang Y, Wang X, et al Species composition and environmental adaptation of indigenous Chinese cattle. Sci Rep. Nature Publishing Group; 2017;7: 16196 10.1038/s41598-017-16438-7 29170422 PMC5700937

[23] Sempéré G, Moazami-Goudarzi K, Eggen A, Laloë D, Gautier M, Flori L. WIDDE: a Web-Interfaced next generation database for genetic diversity exploration, with a first application in cattle. BMC Genomics. BioMed Central; 2015;16: 940 10.1186/s12864-015-2181-1 PMC4647285 26573482

[24] Barbato M, Hailer F, Upadhyay M, Del Corvo M, Colli L, Negrini R, et al Adaptive introgression from indicine cattle into white cattle breeds from Central Italy. Sci Rep. Springer US; 2020;10: 1–11. 10.1038/s41598-019-56847-4 31992729 PMC6987186

[25] Decker JE, Pires JC, Conant GC, McKay SD, Heaton MP, Chen K, et al Resolving the evolution of extant and extinct ruminants with high-throughput phylogenomics. Proc Natl Acad Sci. 2009;106: 18644–18649. 10.1073/pnas.0904691106 19846765 PMC2765454

[26] Chang CC, Chow CC, Tellier LC, Vattikuti S, Purcell SM, Lee JJ. Second-generation PLINK: rising to the challenge of larger and richer datasets. Gigascience. 2015;4: 7 10.1186/s13742-015-0047-8 25722852 PMC4342193

[27] Matukumalli LK, Lawley CT, Schnabel RD, Taylor JF, Allan MF, Heaton MP, et al Development and characterization of a high density SNP genotyping assay for cattle. PLoS One. Public Library of Science; 2009;4: e5350 10.1371/journal.pone.0005350 19390634 PMC2669730

[28] Pitt D, Sevane N, Nicolazzi EL, MacHugh DE, Park SDE, Colli L, et al Domestication of cattle: Two or three events? Evol Appl. 2019;12: 123–136. 10.1111/eva.12674 30622640 PMC6304694

[29] Albrechtsen A, Nielsen FC, Nielsen R. Ascertainment biases in SNP chips affect measures of population divergence. Mol Biol Evol. 2010;27: 2534–2547. 10.1093/molbev/msq148 20558595 PMC3107607

[30] McTavish EJ, Hillis DM. How do SNP ascertainment schemes and population demographics affect inferences about population history? BMC Genomics. 2015;16 10.1186/s12864-014-1191-8 25887858 PMC4428227

[31] Malomane DK, Reimer C, Weigend S, Weigend A, Sharifi AR, Simianer H. Efficiency of different strategies to mitigate ascertainment bias when using SNP panels in diversity studies. BMC Genomics. 2018;19: 22 10.1186/s12864-017-4416-9 29304727 PMC5756397

[32] Ma G, Chang H, Li S, Chen H, Ji D, Geng R, et al Phylogenetic Relationships and Status Quo of Colonies for Gayal Based on Analysis of Cytochrome b Gene Partial Sequences. J Genet Genomics. Elsevier; 2007;34: 413–419. 10.1016/S1673-8527(07)60045-9 17560527

[33] MacEachern S, Hayes BJ, McEwan J, Goddard M. An examination of positive selection and changing effective population size in Angus and Holstein cattle populations (Bos taurus) using a high density SNP genotyping platform and the contribution of ancient polymorphism to genomic diversity in Domestic ca. BMC Genomics. 2009;10: 181 10.1186/1471-2164-10-181 19393053 PMC2681480

[34] Clayton D. snpStats: SnpMatrix and XSnpMatrix classes and methods. 2017.

[35] Davison AC, Hinkley D V. Bootstrap methods and their application. Cambridge, United Kingdom: Cambridge University Press; 1997.

[36] Alexander DH, Novembre J, Lange K. Fast model-based estimation of ancestry in unrelated individuals. Genome Res. 2009;19: 1655–1664. 10.1101/gr.094052.109 19648217 PMC2752134

[37] Huson DH, Bryant D. Application of phylogenetic networks in evolutionary studies. Mol Biol Evol. 2006;23: 254–267. 10.1093/molbev/msj030 16221896

[38] Pickrell JK, Pritchard JK. Inference of Population Splits and Mixtures from Genome-Wide Allele Frequency Data. PLoS Genet. 2012;8 10.1371/journal.pgen.1002967 PMC3499260 23166502

[39] R Core Team. R: a language and environment for statistical computing. Vienna: R Foundation for Statistical Computing [Internet]. Vienna; 2012 Available: http://www.rproject.org

[40] McTavish EJ, Hillis DM. How do SNP ascertainment schemes and population demographics affect inferences about population history? BMC Genomics. BioMed Central; 2015;16: 266 10.1186/s12864-015-1469-5 PMC4428227 25887858

[41] Lachance J, Tishkoff SA. SNP ascertainment bias in population genetic analyses: Why it is important, and how to correct it. Bioessays. 2014;35: 780–786. 10.1002/bies.201300014.SNP PMC3849385 23836388

[42] Utsunomiya YT, Milanesi M, Fortes MRS, Porto-Neto LR, Utsunomiya ATH, Silva MVGB, et al Genomic clues of the evolutionary history of Bos indicus cattle. Anim Genet. 2019;50: 557–568. 10.1111/age.12836 31475748

[43] Zhang W, Gao X, Zhang Y, Zhao Y, Zhang J, Jia Y, et al Genome-wide assessment of genetic diversity and population structure insights into admixture and introgression in Chinese indigenous cattle. BMC Genet. BMC Genetics; 2018;19: 114 10.1186/s12863-018-0705-9 30572824 PMC6302425

[44] Chen N, Cai Y, Chen Q, Li R, Wang K, Huang Y, et al Whole-genome resequencing reveals world-wide ancestry and adaptive introgression events of domesticated cattle in East Asia. Nat Commun. 2018;9: 2337 10.1038/s41467-018-04737-0 29904051 PMC6002414

[45] Lai S-J, Liu Y-P, Liu Y-X, Li X-W, Yao Y-G. Genetic diversity and origin of Chinese cattle revealed by mtDNA D-loop sequence variation. Mol Phylogenet Evol. Academic Press; 2006;38: 146–154. 10.1016/j.ympev.2005.06.013 16054846

[46] Lei CZ, Chen H, Zhang HC, Cai X, Liu RY, Luo LY, et al Origin and phylogeographical structure of Chinese cattle. Anim Genet. John Wiley & Sons, Ltd (10.1111); 2006;37: 579–582. 10.1111/j.1365-2052.2006.01524.x 17121603

[47] Cai X, Chen H, Lei C, Wang S, Xue K, Zhang B. mtDNA Diversity and genetic lineages of eighteen cattle breeds from Bos taurus and Bos indicus in China. Genetica. Springer Netherlands; 2007;131: 175–183. 10.1007/s10709-006-9129-y 17203371

[48] Mohamad K, Olsson M, van Tol HTA, Mikko S, Vlamings BH, Andersson G, et al On the Origin of Indonesian Cattle. DeSalle R, editor. PLoS One. 2009;4: e5490 10.1371/journal.pone.0005490 19436739 PMC2677627

[49] Lawson DJ, van Dorp L, Falush D. A tutorial on how not to over-interpret STRUCTURE and ADMIXTURE bar plots. Nat Commun. 2018;9: 3258 10.1038/s41467-018-05257-7 30108219 PMC6092366

[50] British Army Aid Group. An Outline of Conditions in Occupied Hong Kong [Internet]. 1945. Available: https://industrialhistoryhk.org/world-war-1945-baag-report-occupied-hong-kong-dairy-supplies-facilities/

