# Supplementary material for: A genetically unique Chinese cattle population shows evidence of common ancestry with wild species when analysed with a reduced ascertainment bias SNP panel
Source: PLoS One. 2020 Apr 9;15(4):e0231162. doi: 10.1371/journal.pone.0231162 (PMC7145104; doi:10.1371/journal.pone.0231162)
Supplement: S1 Text — (DOC) [file pone.0231162.s001.doc]

# Supplementary S1 Text

Hong Kong was a British colony for 151 years, from 1842 to 1941 and from 1945 to 1997, and an important transhipment port for British trade with China due to its strategic geographical position [1,2]. Until the 1950’s, the British government promoted local agricultural in Hong Kong to provide the city with rice, vegetables and meat [3]. Reports of the Southern District Officers from 1910 to 1950 described activities in the rural areas of Hong Kong: for each village assessed, the population size and the geographic and demographic description of the animal bred present were reported. According to these reports several villages bred “short horned brown cows” which provided draught power for ploughing, especially on Lantau Island [4]. The use of cattle to plough paddy fields is referred even during Japanese occupation from 1941 to 1945 [5].

The historical background played a significant role in the introduction of other cattle breeds to Hong Kong. Under British control, cattle imports included dairy breeds from Australia, Canada, Scotland, and the Netherlands [6,7]. The dairy breeds import ceased in 1941 during the Japanese occupation and was then resumed in 1945 by the reinstalled British government. Trade relations with China ceased in 1951, when the United Nations imposed an embargo [1,8].

The rapid urbanization and economic development during the 1960s lead to industrialization. The economic competitiveness of local products declined, and over 50% of the farming population abandoned agriculture and moved to the city. As a consequence, starting from 1961 the local agricultural production declined and was not sufficient to supply Hong Kong, which consequently started to depend increasingly on food imports [3]. It is though that farmers who abandoned the agriculture released the cattle into the nearby forests, where they began to roam freely [9–11]. According to a detailed survey conducted by the Hong Kong government in 2013, around 57% of the estimated 1,250 head of feral cattle are considered as “feral herds”, mostly found in the Country Parks of Ma On Shan/Sai Kung, Northeast and Central New Territories and Lantau Island. The remaining 43% are considered “town herds”, mostly distributed on Lantau Island [12]. Complaints are regularly made by the local community regarding cattle, both in terms of traffic disturbance, and damage to natural vegetation and crops impact [13,14]. To tackle the problem non-culling approaches to control the stray cattle populations are currently being evaluated [13,15].

**References**

1. Tao Z, Wong YCR. Hong Kong : From Entrepôt to Manufacturing and Then to Producer Services 1. 2001; 1–12.

2. Schenk C. Economic History of Hong Kong [Internet].

3. Bai X, Wang J, Chow S. Shifting roles and development of agriculture in Hong Kong. Res Rev J Soc Sci. 2015;2: 1–10.

4. Hamilton Eric, Walter S, S.H. P, Tsui P, Coates A, Hayes J. Southern District Officer Reports: Islands and Villages in Rural Hong Kong, 1910-60. Strickland J, editor. 2010.

5. Ho P. Making Hong Kong - A history of its urban development. 2018.

6. Dairy Farm Company – A timeline – The Industrial History of Hong Kong Group [Internet].

7. Industry history - Australian Livestock Exporters’ Council (ALEC) [Internet].

8. Part1 Chpater 6.1 - The embargo and industrialisation [Internet].

9. LegCo Panel on Food Safety and Environmental Hygiene-Strategy adopted by the government in tackling the issue of stray cattle. 2014.

10. Agriculture, Fisheries and Conservation Department - Inspection &amp; Quarantine - Stray Cattle and Buffalo Management Plan [Internet].

11. Dudgeon D, Corlett RT. The ecology and biodiversity of Hong Kong. 2004.

12. Hong Kong Government. LCQ22: Management of stray cattle [Internet]. 2015.

13. Massei G, Koon KK, Benton S, Brown R, Gomm M, Orahood DS, et al. Immunocontraception for managing feral cattle in Hong Kong. PLoS One. 2015;10: 1–14. doi:10.1371/journal.pone.0121598

14. Leung GPC, Hau BCH, Corlett RT. Exotic plant invasion in the highly degraded upland landscape of Hong Kong, China. Biodivers Conserv. 2009;18: 191–202. doi:10.1007/s10531-008-9466-5

15. Massei G, Koon KK, Law SI, Gomm M, Mora DSO, Callaby R, et al. Fertility control for managing free-roaming feral cattle in Hong Kong. Vaccine. Elsevier Ltd; 2018;36: 7393–7398. doi:10.1016/j.vaccine.2018.09.071
