# Supplementary figures and images for: A genetically unique Chinese cattle population shows evidence of common ancestry with wild species when analysed with a reduced ascertainment bias SNP panel
Source: PLoS One. 2020 Apr 9;15(4):e0231162. doi: 10.1371/journal.pone.0231162 (PMC7145104; doi:10.1371/journal.pone.0231162)

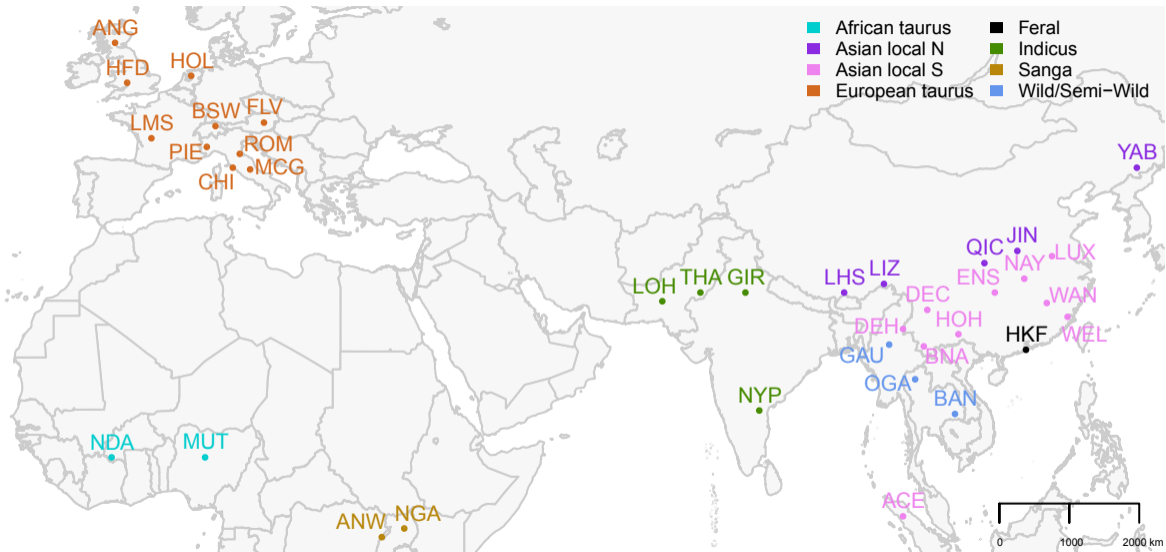

Supplement: S1 Fig — (PDF) [file pone.0231162.s005.pdf]

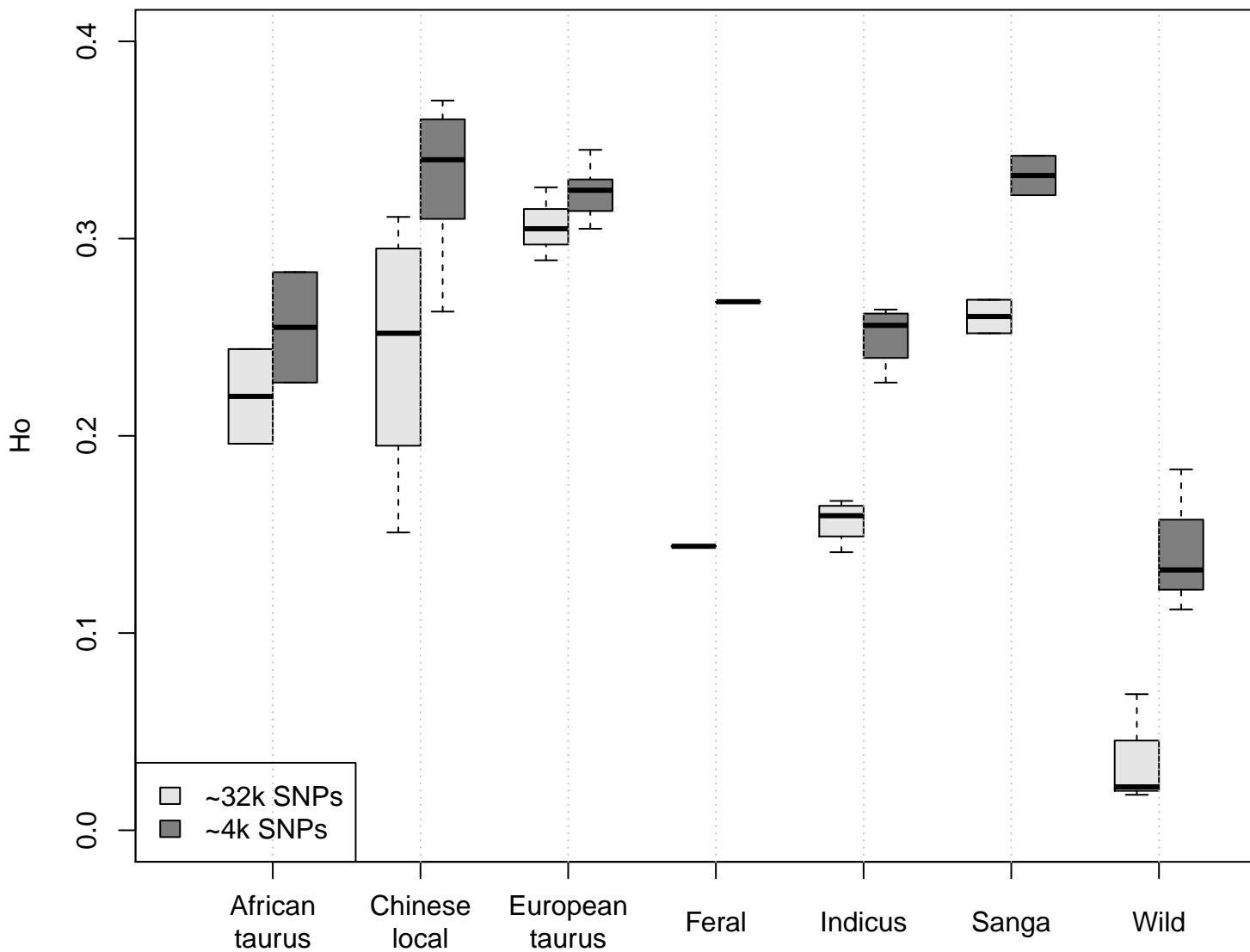

Supplement: S2 Fig — (PDF) [file pone.0231162.s006.pdf]

Ho: 1000 permutations

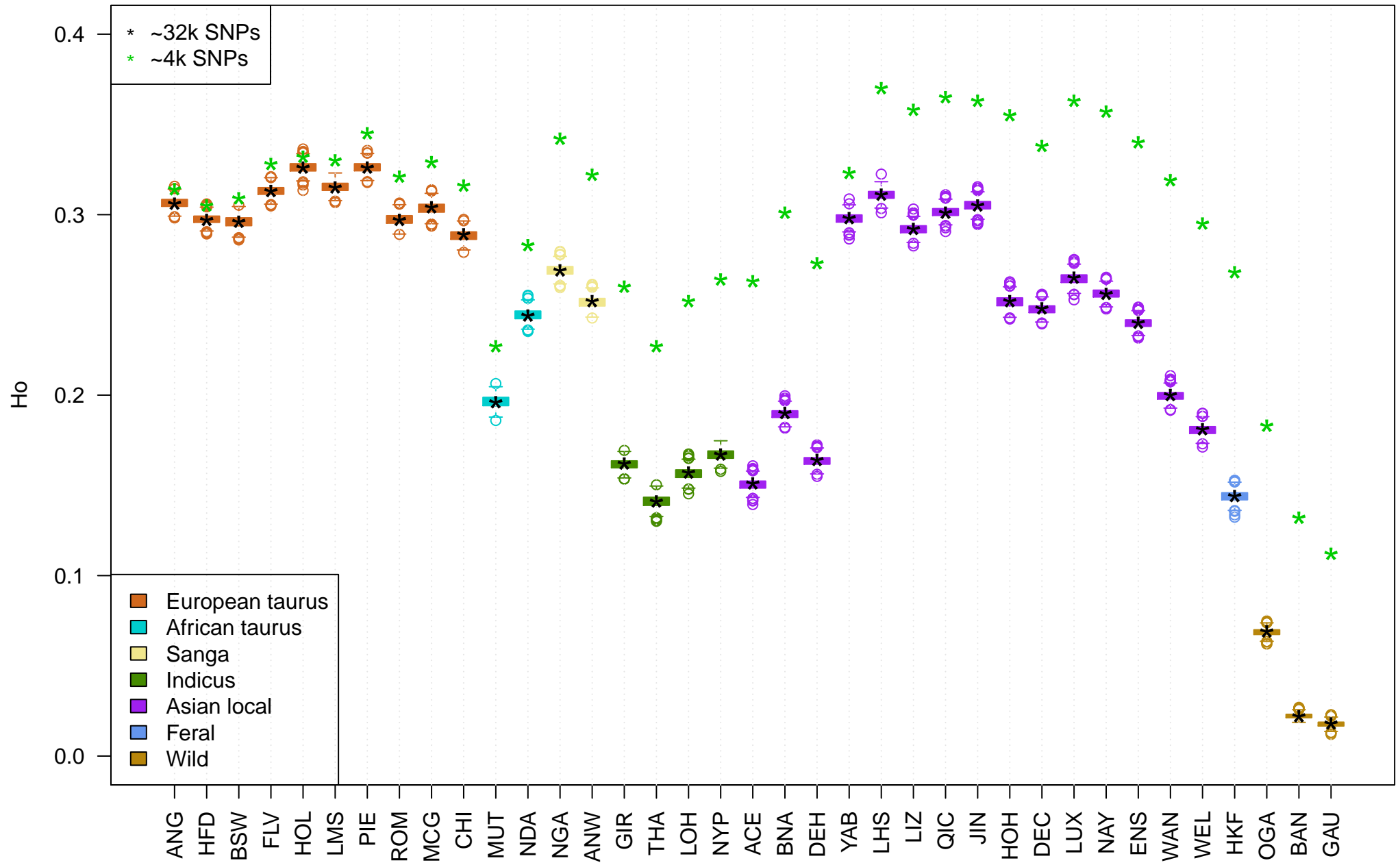

Supplement: S3 Fig — (PDF) [file pone.0231162.s007.pdf]

A)

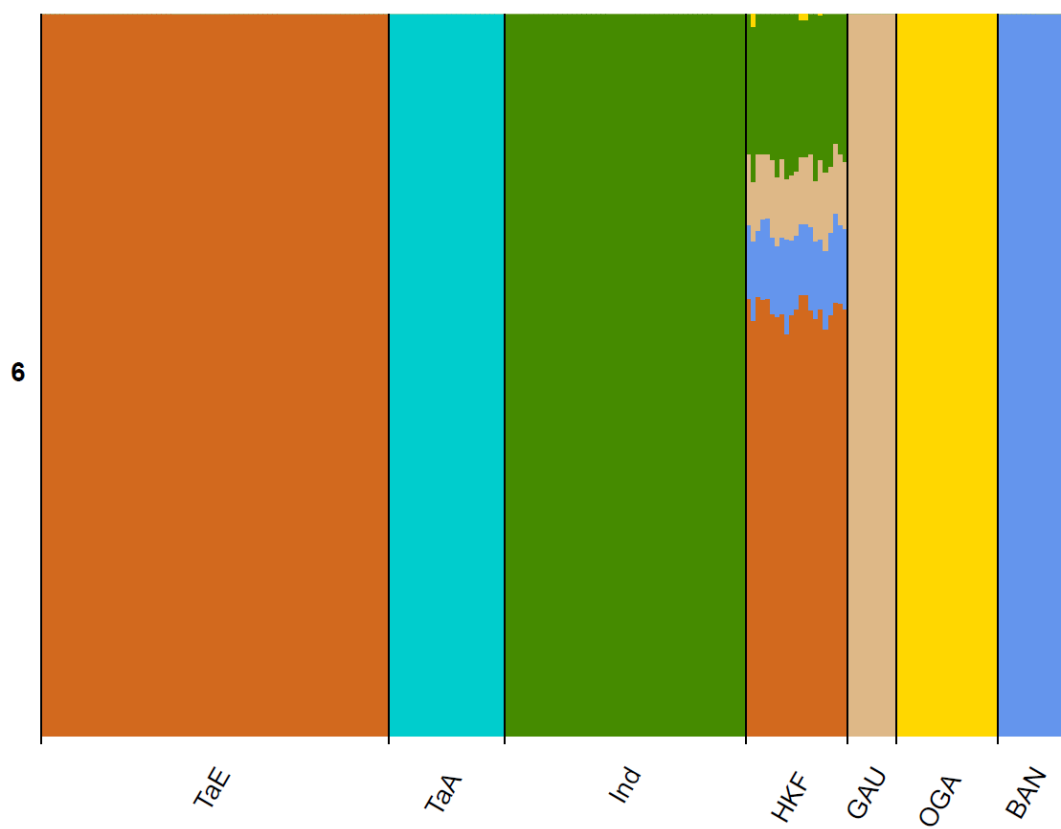

B)

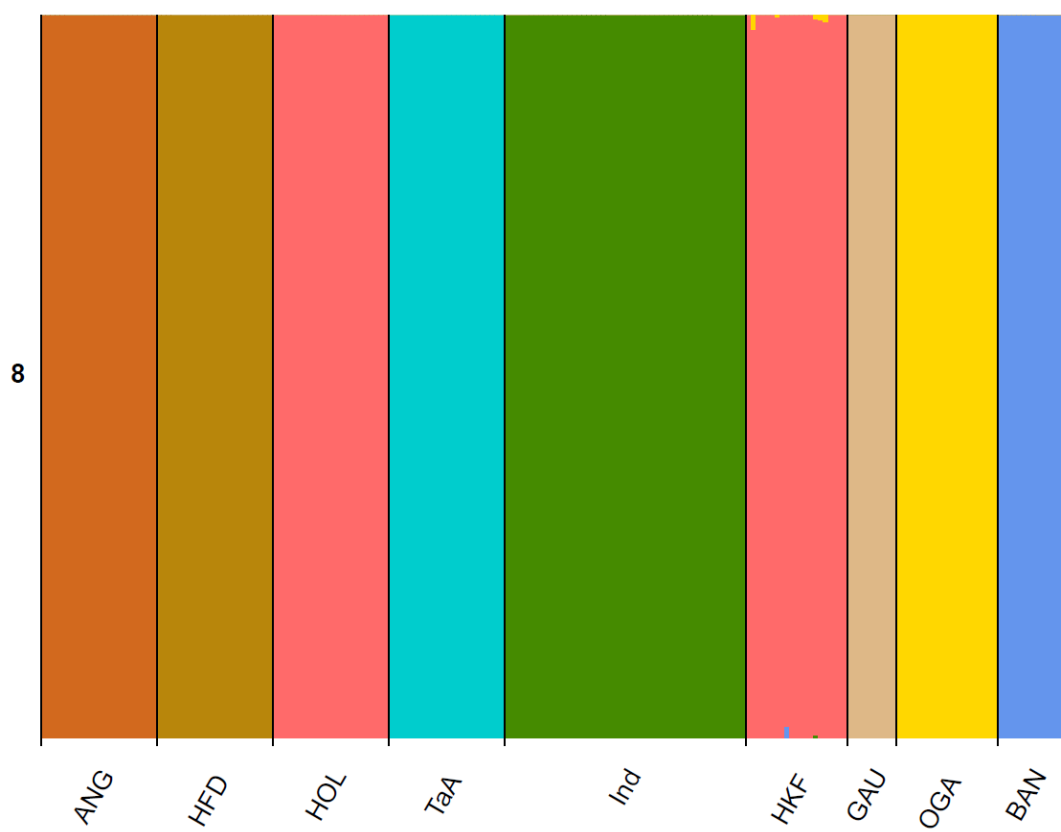

Supplement: S4 Fig — Supervised Admixture analysis of HKF performed with ~4k SNPs using A) a meta-group of taurine references and B) separating the three European taurine breeds as prior populations. The European taurine (TaE) meta-group includes ANG, HFD and HOL, the African Taurine (TaA) includes MUT and the non-admixed individuals of NDA, and the Indicine (Ind) group includes GIR, THA and LOH. (PDF) [file pone.0231162.s008.pdf]

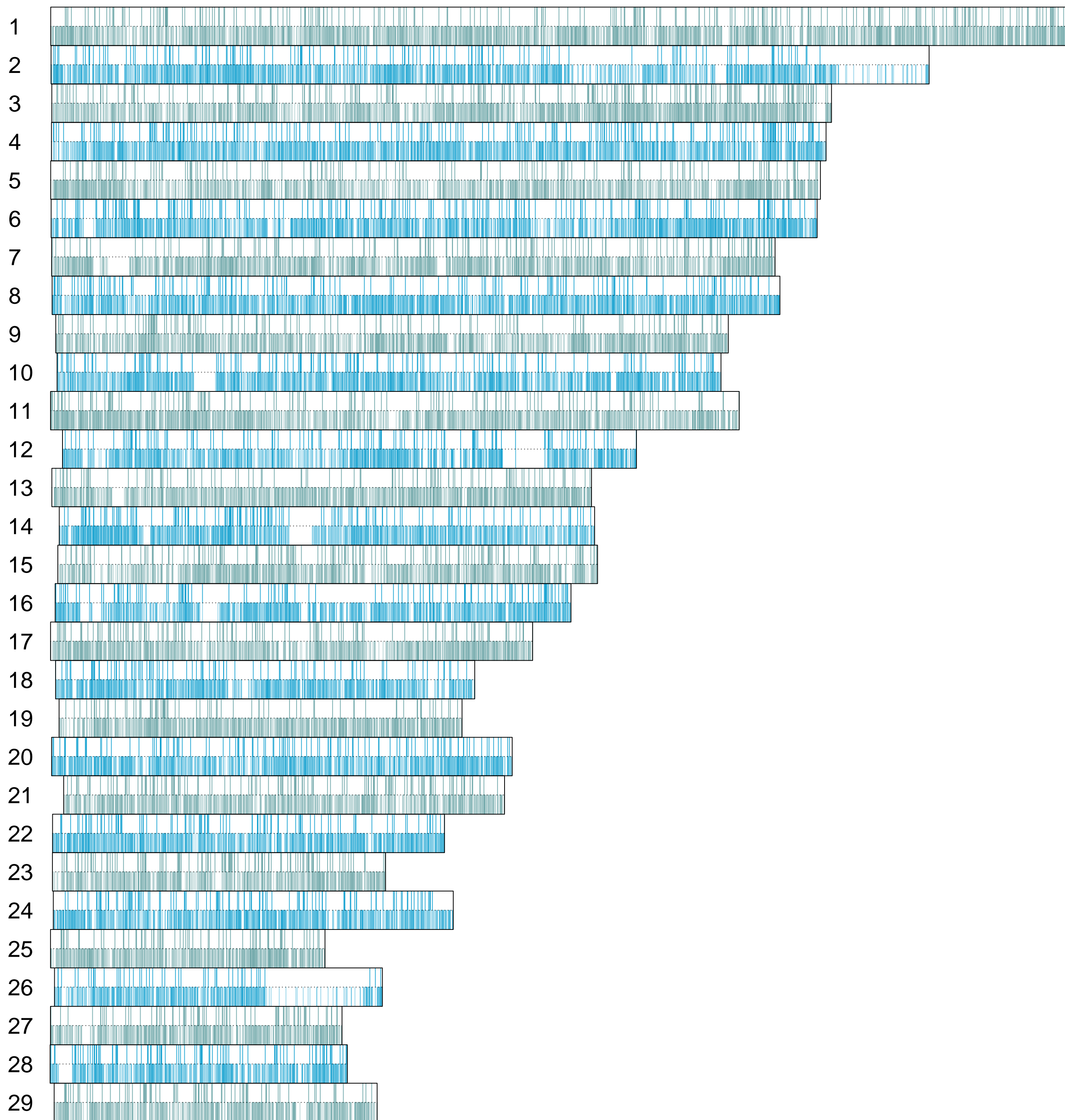

Supplement: S5 Fig — The vertical lines in the upper and lower part of each chromosome schematic represent the SNPs in the ~4k and ~32k dataset, respectively. (PDF) [file pone.0231162.s009.pdf]

A)

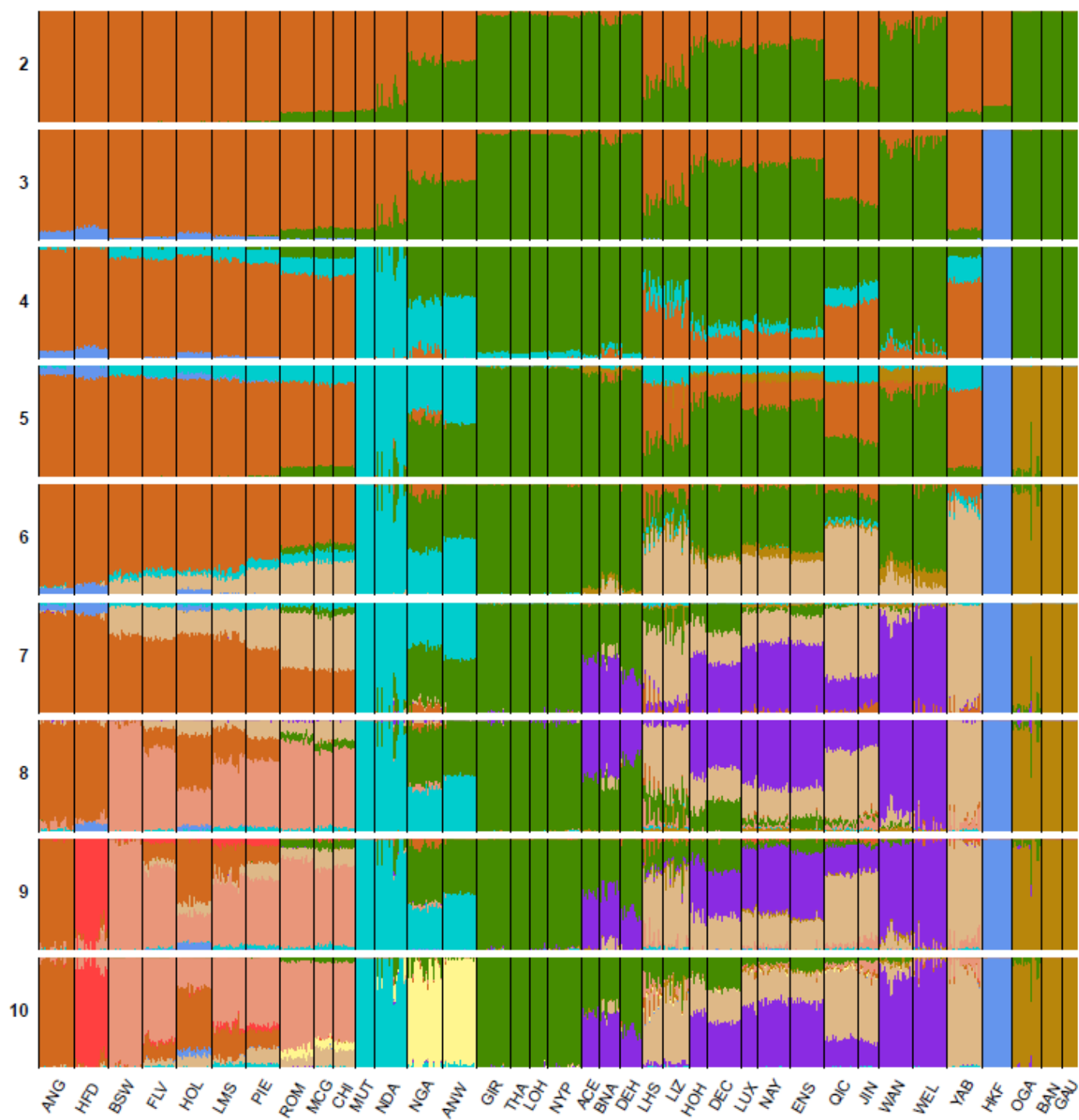

B)

~32k SNPs

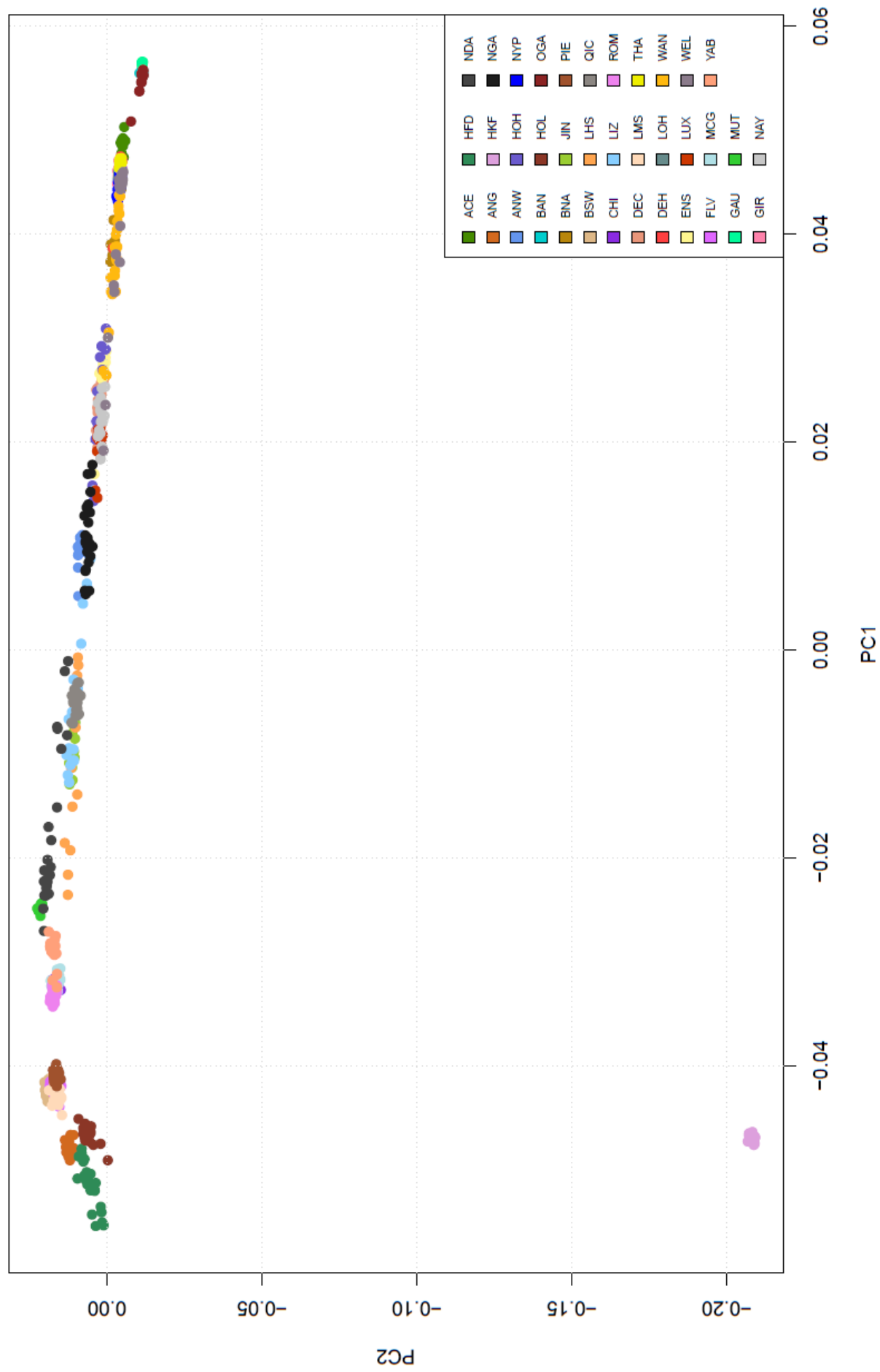

Supplement: S6 Fig — A) Admixture analysis of the first 10 K solutions for 37 cattle populations using ~32k SNPs. B) Principal component analysis using ~32k SNPs. The percentage values within brackets refer to the proportion of variance explained by each of the displayed principal components. For population abbreviations see Table 1. C) Neighbour-Net of Reynold’s distances using ~32k SNPs. (PDF) [file pone.0231162.s010.pdf]
